# Hit me with your best shot: combining gene editing events to deliver iron and zinc biofortified rice

**DOI:** 10.3389/fpls.2026.1878163

**Published:** 2026-09-21

**Authors:** Paloma Koprovski Menguer, Lucas Adamy Lenhardt, Marcos Bönes, Felipe Klein Ricachenevsky

**Affiliations:** 1 Instituto de Tecnologia Química e Biológica António Xavier, Universidade Nova de Lisboa, Oeiras, Portugal; 2 Laboratório de Fisiologia Vegetal, Departamento de Botânica, Universidade Federal do Rio Grande do Sul, Porto Alegre, Brazil; 3 Programa de Pós-Graduação em Botânica, Universidade Federal do Rio Grande do Sul, Porto Alegre, Brazil; 4 Programa de Pós-Graduação em Biologia Celular e Molecular, Centro de Biotecnologia, Universidade Federal do Rio Grande do Sul, Porto Alegre, Brazil

**Keywords:** biofortification, gene editing, iron, rice, zinc

## Abstract

Micronutrient deficiencies, particularly of iron (Fe) and zinc (Zn), are major global health concerns for humans, especially in populations that rely heavily on cereal-based diets. Rice grains, one of the world’s most consumed staple crops, have low concentrations of bioavailable Fe and Zn, especially in the endosperm which constitutes white rice, the most consumed rice. Biofortification is proposed as an excellent cost-effective strategy to solve the problem. Recent advances in CRISPR-based genome editing now allow precise manipulation of genes controlling mineral uptake, transport, sequestration, and grain partitioning, offering new opportunities to generate non-transgenic, biofortified crop. Here we discuss current knowledge on the genetic control of Fe and Zn accumulation in rice grains and evaluate how this information can be leveraged to design effective genome editing strategies for nutritional improvement. We focus on candidate genes involved in vacuolar sequestration, metal chelator biosynthesis and transport, plasma membrane transport, regulatory pathways controlling metal homeostasis, and phytic acid metabolism affecting mineral bioavailability. Evidence from loss-of-function mutants, promoter engineering, natural allelic variation, and precise sequence editing demonstrates that targeted manipulation of these pathways can substantially enhance Fe and Zn accumulation, particularly in polished rice, without major yield penalty. We further propose potential multiplex gene editing approaches to combine alleles and optimize metal allocation to the endosperm. These advances provide a framework for the rational development of next-generation, non-transgenic biofortified rice cultivars.

## Introduction

Hidden hunger is a widespread form of micronutrient deficiency that affects more than two billion people worldwide (Bouis and Saltzman, 2017; Stevens et al., 2022). Of these deficiencies, Fe and Zn are among the most prevalent (Passarelli et al., 2024). Populations relying predominantly on cereal-based staple foods, such as wheat, rice, and maize, are especially vulnerable due to low mineral and high phytate concentration (Cakmak, 2008; White and Broadley, 2009). Although conventional interventions such as supplementation and food fortification can reduce micronutrient deficiencies, they are limited by cost, infrastructure, and the necessary long-term commitment, particularly in low-income regions (Bhutta et al., 2013). In contrast, biofortification has emerged as a cost-effective strategy to increase the concentration of essential micronutrients in edible plant tissues (Bouis and Saltzman, 2017). This approach can be achieved by agronomic management that also depends on constant input; conventional breeding, which needs genetic variation for introgression, which is limited; and modern biotechnological tools such as genetic engineering and genome editing (Saltzman et al., 2013). From these, CRISPR/Cas-based genome editing has emerged as a key tool for precise and efficient changes in crop nutritional traits (Chen et al., 2019).

The CRISPR/Cas9 system uses a guide RNA (gRNA) to direct the Cas9 nuclease to a specific DNA sequence located next to a protospacer adjacent motif (PAM), where it induces a double-strand break (DSB) that is predominantly repaired by non-homologous end joining (NHEJ) in plants. This error-prone pathway frequently generates small insertions or deletions, making CRISPR/Cas9 particularly effective for gene knockouts (Doudna and Charpentier, 2014). To enable more precise genome editing, CRISPR-derived technologies have been developed. Base editing allows direct nucleotide conversions without inducing DSBs, while prime editing combines a Cas9 nickase with a reverse transcriptase to introduce targeted substitutions, insertions, or deletions (Chen and Liu, 2023). In addition, other CRISPR-associated systems, such as Cas12 and Cas13, further expand the editing toolbox by enabling DNA targeting with distinct PAM requirements and programmable RNA targeting, respectively (Zetsche et al., 2017; Zhan et al., 2023).

The ability to generate targeted genetic modifications without the stable integration of foreign DNA has important implications for the regulatory classification and public perception of genome-edited crops. In many regulatory frameworks, gene edition events, particularly those classified as SDN-1 and SDN-2, are distinguished from transgenic approaches, as they result in small insertions or deletions (SDN-1), or precise nucleotide substitutions, insertions, or deletions using a repair template (SDN-2), without introducing exogenous genetic material. Consequently, genome-edited crops may face fewer regulatory constraints, and, in some contexts, greater public acceptance compared to conventional genetically modified organisms (GMOs) (Buchholzer and Frommer, 2023; Groover et al., 2024), making its deployment to the market more straightforward. Given that the technology is mature and biofortification needs to be tackled, our current bottleneck is knowledge about which genetic modifications could be used and combined to deliver biofortified crops. In this Mini-Review, we examine how current knowledge on genes and mutations associated with Fe and Zn accumulation can be leveraged to design non-transgenic biofortification strategies based on gene editing. Given the available literature, we focused primarily on rice (*Oryza sativa* L.) as the major model for cereal biofortification, examining the most promising candidate genes and editing strategies, including loss-of-function and precise sequence modifications, and suggests the best edit combinations to deliver Fe- and Zn-enriched genotypes.

## Candidate genes for rice biofortification

Rice is the second most important staple food crop cultivated worldwide and is consumed by more than half of the global population, accounting for approximately 17% of total caloric intake (Elert, 2014; Fukagawa and Ziska, 2019; FAO, 2023). It is particularly important as it provides up to 50% of the caloric intake for populations living in poverty in Asia, and its consumption is steadily increasing in developing regions of Latin America, the Caribbean, and Africa (Muthayya et al., 2014). The main cultivated species is asian rice, *Oryza sativa* L., which is widely grown worldwide, whereas *Oryza glaberrima* Steud. is cultivated locally in Africa (Muthayya et al., 2014). Current evidence identifies a set of core processes regulating Fe and Zn accumulation in grains, including vacuolar sequestration, chelator biosynthesis, metal–chelate transport, and regulatory control of metal distribution (Kobayashi and Nishizawa, 2012; Ricachenevsky et al., 2015). Importantly, these processes are highly interconnected and collectively shape not only total metal accumulation but also tissue-specific partitioning within the grain, which is critical for nutritional quality given that rice consumption is mostly focused on the endosperm as white rice. Therefore, rice grains biofortification should target accumulation of bioavailable nutrients in this tissue (Stanton et al., 2022; Wairich et al., 2022).

## Vacuolar transporters controlling Fe and Zn delivery to seeds

Vacuolar sequestration represents an important level of control over metal partitioning (Ricachenevsky et al., 2018). Early work has shown that vacuolar iron transporters OsVIT1 and OsVIT2 sequester Fe and Zn into vacuoles and are highly expressed in flag leaves, where they limit metal remobilization to developing grains (Zhang et al., 2012). Loss of function of either gene increases source-to-sink flux, elevating Fe (1.3–1.4-fold) and Zn (1.2–1.6-fold) concentrations in brown rice, particularly in the embryo, while reducing in flag leaves (Zhang et al., 2012). *OsVIT2* was also shown to be expressed in other tissues, such as nodes, the leaf mestome sheath and aleurone layer of the caryopsis (Che et al., 2021). CRISPR/Cas9-mediated knockout of *OsVIT2* increases polished rice Fe concentration (1.4–1.8-fold) without yield penalty (Che et al., 2021). Further work showed that *OsVIT2* is the likely causal gene of EMS-generated mutant 1095_k, which accumulates more Fe (~1.4-fold) and Zn (~1.2-fold) compared to WT in field trials, also without yield penalty (Kandwal et al., 2022).

Since single mutants for *OsVIT1* and *OsVIT2* results in increased nutrient concentration in seeds, recent work tested whether combining *OsVIT1* and *OsVIT2* loss-of-function mutations would have additive effects. Although *osvit1osvit2* double mutants were not directly compared to single mutants *osvit1* and *osvit2* in the same experimental design, Fe and Zn concentrations in seeds were increased in *osvit1osvit2* mutants to similar levels compared to the reported concentrations in single mutants (Zhang et al., 2012; Che et al., 2021). However, *osvit1osvit2* were clearly more sensitive to Fe overload, suggesting that loss of both VIT transporters may result in a trade-off between biofortification and Fe toxicity tolerance (Benato et al., 2026). Thus, this work demonstrates that careful testing for Fe excess tolerance must be considered in biofortification approaches involving VIT genes in rice, given that Fe excess can be a problem in waterlogged or rainfed rice systems (Wairich et al., 2024).

The vacuolar Zn transporter OsMTP1 sequesters Zn into vacuoles and is preferentially expressed in roots, as well as in the aleurone layer and embryo of developing seeds (Ning et al., 2023). *OsMTP1* loss of function reduces Zn concentration in these tissues, increasing root-to-shoot translocation and enhancing Zn accumulation in polished rice (1.3 to 1.4 fold–) without yield penalty. *OsMTP1* knockout also decreases Cd accumulation in grain, indicating a dual benefit for biofortification. Haplotype analysis further revealed alleles associated with reduced *OsMTP1* expression and increased Zn accumulation in polished rice, supporting the potential of natural regulatory variation to fine-tune vacuolar sequestration, increasing Zn while decreasing Cd accumulation in grains (Ning et al., 2023). Together, these findings identify OsMTP1 as a key regulator of vacuolar Zn storage and intra-grain distribution, and a promising target for biofortification.

In addition to direct metal sequestration, vacuoles also regulate the availability of metal chelators. The tonoplast-localized transporter OsVMT regulates Fe and Zn translocation by sequestering the phytosiderophore deoxymugineic acid (DMA) into vacuoles, thereby limiting chelator availability for long-distance transport (Che et al., 2019). Notably, *OsVMT* is highly expressed in node I, a key anatomical hub for metal redistribution to the grain, particularly in the parenchyma cell bridges that mediate intervascular transfer of metals. *OsVMT* loss of function increases cytosolic DMA, enhancing Fe–DMA and Zn–DMA complex formation and promoting metal flux toward developing grains. *osvmt* plants have increased Fe (~2-fold) and Zn (~1.5-fold) accumulation in polished rice and no yield penalty (Che et al., 2019), identifying *OsVMT* as another promising target for biofortification.

## Nicotianamine synthesis and Fe and Zn transport to seeds

Nicotianamine synthase genes (NAS) are key for nicotianamine (NA) biosynthesis, a central Fe and Zn chelator required for long-distance transport. Several studies showed that overexpression or enhanced expression of NAS genes resulted in higher Fe and Zn concentration in seeds (Lee et al., 2009; Johnson et al., 2011; reviewed by Wairich et al., 2022). Given that Fe and Zn are chelated by NA in the phloem, increased expression of NAS genes results in increased Fe and Zn translocation to developing seeds (Johnson et al., 2011). Genome-wide association (GWAS) identified *OsNAS3* within a stable QTL for grain Zn concentration, with distinct promoter haplotypes associated with variation from 18 to 30 mg kg^-1^ Zn in brown rice (Cu et al., 2021). Consistent with this, CRISPR/Cas9-mediated editing of the *OsNAS2* promoter at the ARR1AT cytokinin-responsive cis-element enhanced gene expression, Zn uptake and translocation, and increased Fe and Zn concentrations in brown rice by up to 1.48-fold and 1.23-fold, respectively, while also improving total Zn accumulation per plant and yield-related traits (Ludwig et al., 2024). These findings indicate that promoter engineering of NAS genes, including the recreation of favorable *OsNAS3* haplotypes by gene editing, is a promising strategy to enhance chelator-mediated metal translocation and improve grain Zn accumulation.

## Plasma membrane transporters

The metal–chelate transporter OsYSL9 mediates internal Fe translocation within developing rice grains by transporting Fe(II)–nicotianamine and Fe(III)–deoxymugineic acid complexes. Its expression is strongly and transiently induced during grain-filling stage in the scutellum of the embryo and in endosperm cells surrounding the embryo, where it facilitates Fe translocation from the endosperm to the embryo (Senoura et al., 2017). RNAi-mediated knock-down of *OsYSL9* disrupts this process, resulting in decreased Fe accumulation in embryos but increased Fe in the residual parts of brown and polished seeds. Fe concentration in polished grains is increased by more than twofold, indicating enhanced Fe retention in the endosperm (Senoura et al., 2017). These findings identify *OsYSL9* as a key determinant of intra-grain Fe distribution and a promising target for endosperm biofortification. Notably, *OsYSL9*-RNAi plants were more sensitive to Fe deficiency (Senoura et al., 2017), suggesting that there might a trade-off in suppressing *OsYSL9* expression, which should be taken into account.

OsZIP10 is a plasma membrane-localized transporter highly expressed in the phloem of the flag leaf sheath, as well as in the xylem and vascular bundle sheath of node I and developing grains, and promotes Zn root-to-shoot translocation and distribution to the grain (Zhan et al., 2026). *OsZIP10* loss-of-function impairs Zn distribution and increases sensitivity to Zn deficiency, whereas overexpression enhances Zn accumulation in the grain without yield penalty or increased Cd accumulation. Natural variation in the *OsZIP10* promoter underlies substantial differences in grain Zn content. A single nucleotide polymorphism (A-to-G) at position s21823413 within a B1-like cis-regulatory motif enhances the binding affinity of F-bZIP transcription factors (OsbZIP48/49/50), which control Zn deficiency responses (Hu et al., 2024), resulting in increased *OsZIP10* expression and higher Zn accumulation in brown rice (~1.7-fold) (Zhan et al., 2026). Therefore, *OsZIP10* promoter engineering is another interesting target to increase Zn concentrations.

## Regulatory proteins

HRZ proteins act as negative regulators of Fe homeostasis, functioning as Fe- and Zn-binding ubiquitin ligases that coordinate systemic responses to Fe availability (Kobayashi et al., 2013; Selote et al., 2014). In rice, OsHRZ1 and OsHRZ2 repress Fe deficiency responses by promoting the degradation of positive regulators of Fe uptake and translocation, functioning as a negative feedback control to limit the response to low Fe. Consistent with this role, OsHRZ knockdown lines show constitutive activation of Fe acquisition pathways and increased Fe accumulation in shoots and grains (Kobayashi et al., 2013). Introducing frameshift and in-frame mutations in key functional domains, such as the C-terminal RING Zn-finger domain and rubredoxin-type fold, reduced OsHRZ functional activity and lead to derepression of Fe uptake pathways, thereby enhancing tolerance to Fe deficiency and increasing grain Fe accumulation. Mutations in *OsHRZ1* showed the strongest effect (1.9–2.1-fold) in grain Fe concentration, whereas *OsHRZ2* mutants exhibited a moderate increase (1.5–1.6-fold) (Shinkawa et al., 2025). These findings indicate that modulation of HRZ activity represents a promising strategy for Fe biofortification through genome editing.

## Increasing bioavailability

Targeting phytic acid (PA) biosynthesis has been explored as a strategy to improve micronutrient bioavailability in rice grains. Phytic acid (myo-inositol hexakisphosphate, IP6) accounts for up to 65–85% of seed phosphorus and strongly chelates Fe and Zn, thereby limiting their bioavailability (Lott1 et al., 2000; Raboy, 2003). Grain PA content in rice shows substantial natural variation (0.3–2.98%), reflecting strong genetic control (Tp et al., 2022). This variation has been associated with allelic differences in genes related to phosphorus transport and allocation, including *OsPT8* and *OsSPDT* (Jia et al., 2011; Tp et al., 2022; Fang et al., 2025). In particular, polymorphisms in the 3′ untranslated region of *SPDT* and coding variation in *OsPT8* explain up to 7.84% and 18.49% of phenotypic variation, respectively (Tp et al., 2022). Functional studies further show that disruption of PA biosynthesis genes, such as *OsITPK6* and *OsIPK1*, reduces PA accumulation and increases inorganic phosphorus in grains, but often impairs growth and seed development (Jiang et al., 2019; Wang et al., 2024). Together, these findings suggest that allele-specific genome editing may provide a strategy to fine-tune PA levels in rice grains to improve Fe and Zn bioavailability. However, attempts to alter PA levels should be carefully evaluated, given that reduced PA may alter seed quality, germination and seedling vigor.

Altogether, these findings highlight that Fe and Zn accumulation in rice grains can be tuned by targeting key proteins controlling metal flux and partitioning. Targeted mutations in critical functional domains, gene knockouts, and cis-regulatory modifications represent complementary strategies to modulate these pathways, enabling the optimization of micronutrient allocation to the edible endosperm. This framework provides a basis for the rational development of biofortified rice and its extension to other cereal crops.

## Best combinations for generating non-transgenic, Fe and/or Zn biofortified rice plants

Micronutrient accumulation in cereal grains results from the coordinated action of multiple interconnected pathways, including metal acquisition, transport, storage, and intra-grain redistribution (Kong et al., 2022; Wairich et al., 2022). As such, single-gene interventions are often insufficient to achieve substantial improvements in grain Fe and Zn content (White and Broadley, 2009; Bouis and Saltzman, 2017). Multiplex CRISPR genome editing enables the simultaneous targeting of key regulatory and functional nodes within this network, offering a strategy to fine-tune metal flux and partitioning to target tissues more precisely (Chen et al., 2019; Zhang et al., 2021). A recent study in rice demonstrated that a modular multiplex prime-editing system based on the PE3 strategy can simultaneously target up to four genomic loci in a single generation. PE3 combines an nCas9–reverse transcriptase (nCas9–RT) with a prime-editing guide RNA (pegRNA), which specifies the target and encodes the desired edit, and a nicking guide RNA (ngRNA), which nicks the opposite DNA strand to favor incorporation of the edited sequence. The modular system enables multiple pegRNA–ngRNA units to be assembled into a single binary vector, allowing precise nucleotide substitutions, small insertions and deletions, and functional knockouts to be generated simultaneously at multiple loci (Gupta et al., 2024). This modular system can therefore generate SDN-2 edits, including gene knockouts through targeted deletions/insertions and regulatory element modifications through precise nucleotide substitutions. Importantly, following segregation of the editing machinery, the resulting plants retain only targeted sequence modifications and would not be considered transgenic in regulatory frameworks that exempt genome-edited crops lacking foreign DNA. Building on this strategy, we propose multiplex editing designs to optimize gene combinations for increasing Fe and Zn accumulation in rice seeds, particularly in the endosperm.

Multiplexing pegRNAs with ngRNAs is feasible, and therefore might be faster for generation of edited lines, although independent, single edited lines could be produced and then crossed, allowing testing of lower-order mutants. Importantly, once generated, these lines should be carefully tested in comparison to wild type lines for some traits: (1) Fe/Zn concentration in brown and white rice, ensuring biofortification of the endosperm; (2) altered Fe and Zn distribution in embryos, which can have consequences for germination and seedling viability/vigor; (3) Fe and Zn deficiency, and Fe excess sensitivity determination in controlled conditions in order to guarantee the absence of trade-offs; (4) yield penalty determination in field conditions, including excessive Fe conditions that mimic what is observed in environments in which rice is cultivated (Wairich et al., 2024). For that, we selected the four most promising candidate genes of the single mutants reported in the literature (**Supplementary Table 1**). Our criteria for candidate gene/mutation selection was based on the gene mutation having (1) a positive effect on Fe/Zn concentration; (2) absence of yield reporter seed yield penalty; (3) the possibility of recapitulating such mutation via gene editing, allowing generation of non-transgenic biofortified plants.

For increasing Fe concentration, we propose a combination of SDN-2 edits using the PE3 strategy. Targeted deletions will be introduced in *OsVIT2*, *OsVMT*, and *OsYSL9* to generate gene knockouts, while a targeted single-base-pair deletion within the RING Zn-finger domain of *OsHRZ1* (nucleotides 3376–3416) will be introduced to reduce its activity (**Figure 1**). Together, these edits are expected to increase Fe uptake and long-distance translocation by reducing *OsHRZ1* ubiquitin ligase activity (Shinkawa et al., 2025); lower Fe retention in vacuoles where *OsVIT2* and/or *OsVMT* are expressed, and consequently increased translocation to seeds (Che et al., 2019, 2021); and increase Fe accumulation in the endosperm because of *OsYSL9* loss of function (Senoura et al., 2017). Potential trade-offs should be considered for this combination. Enhanced Fe acquisition resulting from *OsHRZ1* modification may increase susceptibility to Fe toxicity under flooded conditions, where Fe availability is naturally elevated (Wairich et al., 2024). Conversely, *OsYSL9*-knockdown has been associated with increased sensitivity to Fe deficiency, a phenotype that could be partially mitigated by the enhanced Fe uptake expected from *OsHRZ1* modification. In addition, disruption of *OsYSL9* is expected to increase Fe retention in the endosperm while reducing Fe allocation to the embryo, potentially affecting germination and early seedling development. We expect that such multiplex strategy will result in substantially higher Fe concentration compared to single gene mutation, which essentially cause ˜2-fold increase in brown rice and ˜1.5-fold increase in white rice (**Supplementary Table 1**). Still, predicting achievable concentrations is not possible given our current knowledge.

**Figure 1:**
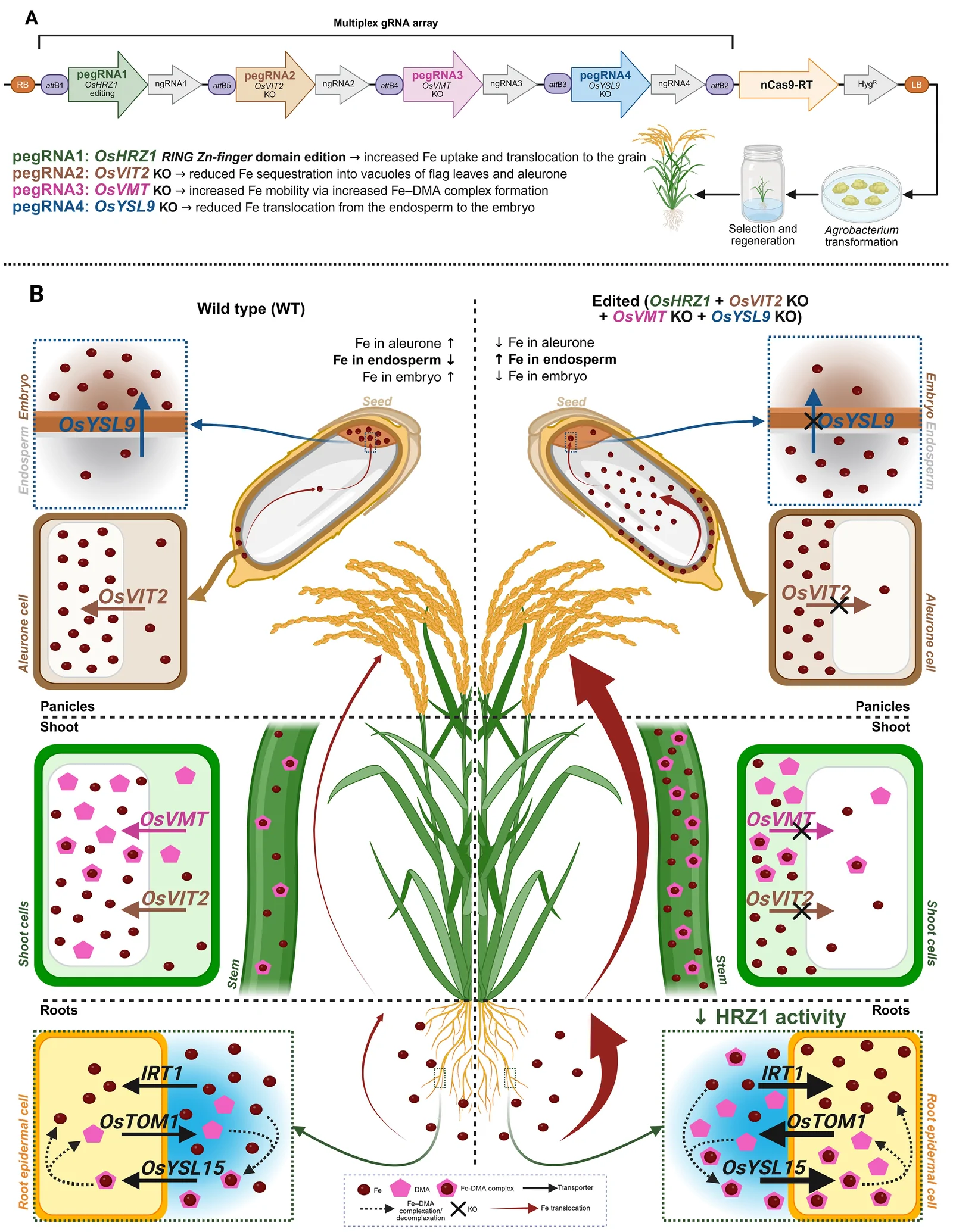
Multiplex prime-editing strategy for iron biofortification in rice. **(A)** Vector ready for Agrobacterium/bombardment-mediated plant transformation for prime editing of up to four genes. The cassette contains four pegRNA/ngRNA modules targeting *OsHRZ1*, *OsVIT2*, *OsVMT*, and *OsYSL9*, followed by the nCas9-RT editor and the HygR selectable marker. Promoters and terminators were omitted for clarity. RB and LB indicate right and left T-DNA borders, respectively. **(B)** Model of Fe redistribution in wild-type (left) and proposed edited plants (right). In wild-type plants, Fe is partially sequestered in shoots, the aleurone layer, and embryo. In edited plants, reduced OsHRZ1 ubiquitin ligase activity is expected to enhance Fe uptake and long-distance translocation, while knockout of *OsVIT2* and *OsVMT* decreases vacuolar Fe sequestration in vegetative tissues and promotes Fe allocation to developing grains. In addition, *OsYSL9* loss of function is proposed to limit Fe transport from the endosperm to the embryo, thereby increasing Fe accumulation in the endosperm. Red circles represent Fe ions, pink hexagons represent DMA molecules, and their association represents Fe–DMA complexes. Arrows indicate Fe transport direction, with thickness proportional to flux intensity; dotted arrows indicate Fe–DMA complexation/decomplexation processes in roots; and crossed arrows indicate gene knockout. Created with Biorender.com.

For increasing Zn concentration, we also propose a combination of SDN-2 edits. Targeted deletions will be introduced in *OsVIT2*, *OsVMT*, and *OsMTP1* to generate gene knockouts, while site-specific editing of the *OsZIP10* promoter will introduce the favorable A-to-G substitution at position s21823413 (**Figure 2**). These edits are expected to decrease Zn retention in *OsVIT2*, *OsMTP1* and/or *OsVMT* expressing tissues (Che et al., 2019, 2021; Ning et al., 2023), while increased *OsZIP10* expression enhances Zn root-to-shoot translocation and distribution to the grain (Zhan et al., 2026). Potential trade-offs should be considered, including the possible accumulation of hazardous trace elements, which requires quantification. Carefully testing Zn deficiency conditions throughout development, including reproductive stages, is also necessary to detect possible increased sensitivity to low Zn. Synergistic effect of multiple gene mutations in seed concentrations is also expected for this strategy, but precise prediction of achievable levels is also not possible.

**Figure 2:**
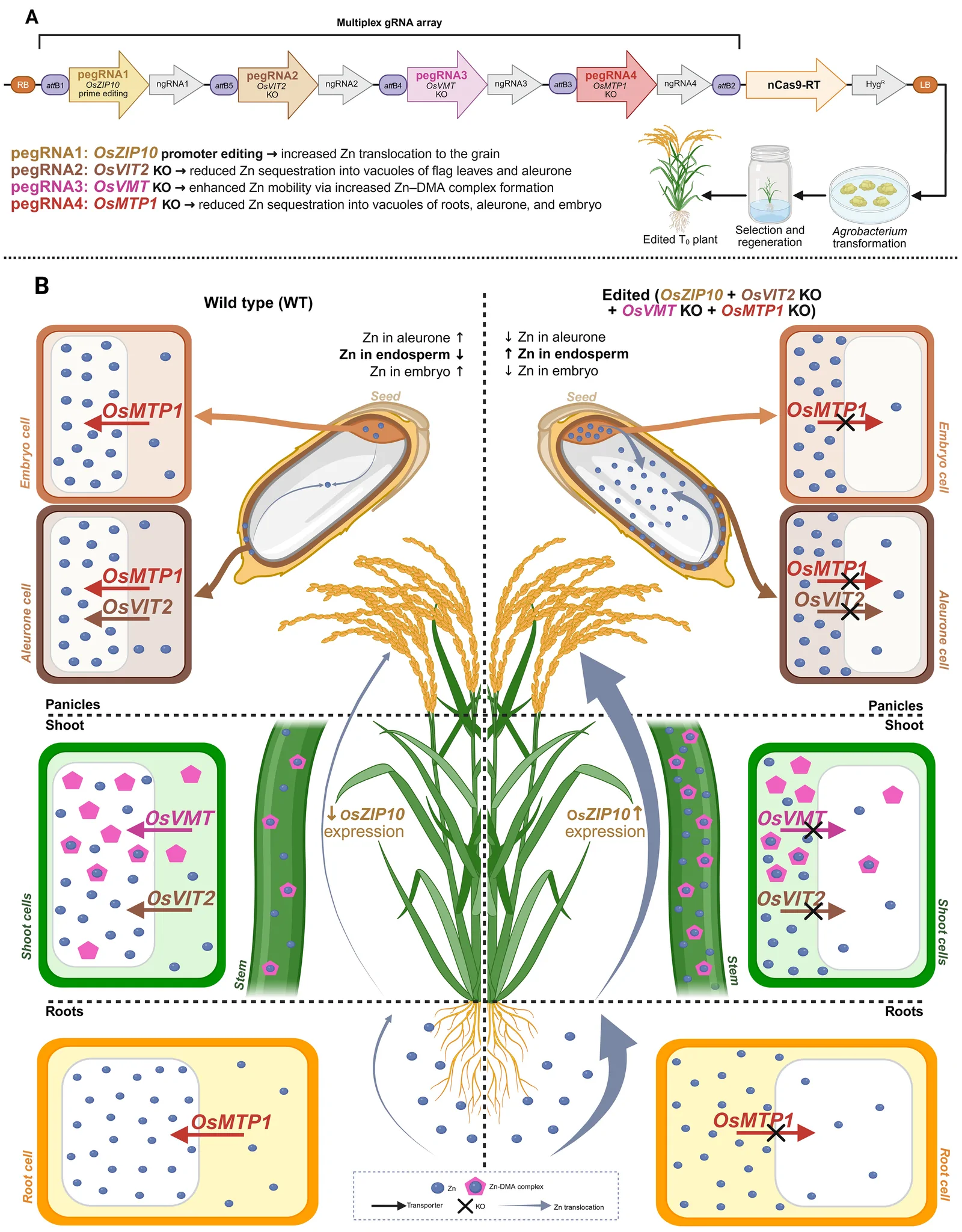
Multiplex prime-editing strategy for zinc biofortification in rice. **(A)** Final vector ready for Agrobacterium/bombardment-mediated plant transformation for prime editing of up to four genes. The cassette contains four pegRNA/ngRNA modules targeting *OsZIP10*, *OsVIT2*, *OsVMT*, and *OsMTP1*, followed by the nCas9-RT editor and the HygR selectable marker. Promoters and terminators were omitted for clarity. RB and LB indicate right and left T-DNA borders, respectively. **(B)** Model of Zn redistribution in wild-type (left) and proposed edited plants (right). In wild-type plants, Zn is partially sequestered in roots, shoots, the aleurone layer, and embryo, limiting its accumulation in the endosperm. In edited plants, increased *OsZIP10* expression enhances root-to-shoot Zn translocation and distribution to developing grains, while knockout of *OsVIT2*, *OsVMT*, and *OsMTP1* reduces vacuolar Zn sequestration in vegetative tissues, increasing Zn accumulation in the endosperm. Blue circles represent Zn ions, pink hexagons represent DMA molecules, and their association represents Zn–DMA complexes. Arrows indicate Zn transport direction, with thickness proportional to flux intensity; dotted arrows indicate Zn–DMA complexation/decomplexation processes in roots; and crossed arrows indicate gene knockout. Created with Biorender.com.

## Future directions

As discussed here, practical biofortification efforts draw from basic science. Thus, further detailed functional gene characterization in rice is necessary to improve the gene toolkit that can be used. Importantly, there is growing information derived from other crops, such as maize (Yan et al., 2023; Shi et al., 2026), sorghum (Liao et al., 2026) and wheat (Harrington et al., 2023), which can be transferred to rice by identification of orthologs. On the other hand, testing the effects of rice orthologs on these Poaceae crops might as well be successful, highlighting the usefulness of rice as a model. Importantly, all biofortification approaches need to encompass comprehensive analyses of modified genotypes in varying metal concentrations, to identify possible trade-offs that could hamper the deployment of cultivars, as well as comparative analyses with other promising genotypes already available in the literature. Thus, researchers in biofortification could benefit from easier distribution of such genotypes among groups. Moreover, it is important to always consider how mutations affect field performance and yield, which is key for farmers’ acceptance, as well as how other nutrients accumulation is altered. Finally, it should be standard for studies in biofortification to also address accumulation of hazardous elements, such as Cd and As, which can accumulate in rice grains, and may be the downside of specific approaches. With that, the community could step closer to delivering biofortified crops that reach the consumer.
