# Supplementary material for: Hit me with your best shot: combining gene editing events to deliver iron and zinc biofortified rice
Source: Front Plant Sci. 2026 Sep 21;17:1878163. doi: 10.3389/fpls.2026.1878163 (PMC13637165; doi:10.3389/fpls.2026.1878163)
Supplement: Supplementary file 1 [file Table1.docx]

**Supplemental Table 1. Candidate genes reported in the literature for which gene editing results in increased Fe and/or Zn concentrations in rice seeds.**

| **Gene** | **Locus ID^A^** | **Genetic Modification /Variation** | **SDN1 or SDN2^B^** | **Fe Effect** | **Zn Effect** | **Yield Penalty** | **Trade-offs reported** | **Reference** | **Notes to justify paralog selection** |
| --- | --- | --- | --- | --- | --- | --- | --- | --- | --- |
| OsVIT1 | LOC_Os04g38940 | Loss-of-function | SDN-1 | ↑ 1.3–1.4× (brown rice) | ↑ 1.2–1.6× (brown rice) | Not reported | Cd²⁺ overaccumulation in brown rice ( plants grown in Cd-contaminated paddy field) | Zhang et al., 2012 |  |
| **OsVIT2** | **LOC_Os09g23300** | Loss-of-function | SDN-1 | ↑ 1.4–1.8× (polished rice)* | ↑ 1.2–1.6× (brown rice)** | No*/ Not reported ** | Cd²⁺ overaccumulation in brown rice ( plants grown in Cd-contaminated paddy field)** | Che et al., 2021*; Zhang et al., 2012** | OsVIT2 was selected as a target given the availability of four papers suggesting positive effects on Fe concentration in knockdown/knockout osvit2 plants, including gene edited lines, while OsVIT1 positive effect on Fe concentrations is supported by only one work, and osvit1osvit2 double mutants are more sensitive to Fe overlad. |
| OsMTP1 | LOC_Os05g03780 | Loss-of-function | SDN-1 | — | ↑ 1.3–1.4× (polished rice) | No | — | Ning et al., 2023 |  |
| **OsVMT** | **LOC_Os12g03899** | Loss-of-function | SDN-1 | ↑ ~2.0× (polished rice) | ↑ ~1.5× (polished rice) | No | — | Che et al., 2019 |  |
| OsNAS3 | LOC_Os07g48980 | Allelic variation | None | — | ↑ 1.67× (brown rice) | No | — | Cu et al., 2021 |  |
| OsNAS2 | LOC_Os03g19420 | Cis-regulatory mutagenesis | SDN-1 | ↑1.46–1.48× (brown rice) | ↑1.17–1.23× (brown rice) | No | — | Ludwig et al., 2023 |  |
| **OsYSL9** | **LOC_Os04g45860** | Loss-of-function | SDN-1 | ↑ >2× (polished rice) | — | Not reported | Potential Fe-deficiency sensitivity | Senoura et al., 2017 |  |
| OsZIP10 | LOC_Os05g39550 | Cis-regulatory mutagenesis/ Allelic variation | SDN-1/none | — | ↑ ~1.7× (brown rice) | No | — | Zhan et al., 2026 |  |
| **OsHRZ1** | **LOC_Os01g49470** | Functional domain mutagenesis | SDN-1 | ↑ 1.9–2.1× (brown rice) | — | Not reported | — | Shinkawa et al., 2025 | OsHRZ1 was selected over OsHRZ2 given that mutations in OsHRZ1 resulted in more pronounced Fe concentration increase compared to OsHRZ2. |
| OsHRZ2 | LOC_Os05g47780 | Functional domain mutagenesis | SDN-1 | ↑ 1.5–1.6× (brown rice) | — | Not reported | — | Shinkawa et al., 2025 |  |
| OsPT8 | LOC_Os10g30790 | Allelic variation | None | Indirect (lower phytic acid) | Indirect (lower phytic acid) | No | — | Tp et al., 2022 |  |
| OsSPDT | LOC_Os06g05160 | Allelic variation | None | Indirect (lower phytic acid) | Indirect (lower phytic acid) | No | — | Tp et al., 2022 |  |
| OsITPK6 | LOC_Os09g34300 | Loss-of-function | SDN-1 | Indirect (lower phytic acid) | Indirect (lower phytic acid) | Not specified | Growth penalty | Jiang et al., 2019 |  |
| OsIPK1 | LOC_Os04g56580 | Loss-of-function | SDN-1 | Indirect (lower phytic acid) | Indirect (lower phytic acid) | Not specified | Growth penalty | Wang et al., 2024 |  |

^A^Gene names and ID number in bold were selected for Fe biofortification; genes names and ID number subscribed were selected for Zn biofortification.

^B^SDN-1, SND-2 or none: mutation/allelic variation described in the original work.
